# Prognosis in Cirrhosis of the Liver

**Published:** 1892-03-26

**Authors:** 


### PROGNOSIS IN CIRRHOSIS OF THE LIVER.

It has been stated with respect to this disease that "it is rare for a patient to live more than a twelvemonth after the symptoms have become so pronounced as to allow a diagnosis to be made." Probably this represents the course of the most common form of the disease in the majority of cases, but there are numerous varieties arising from totally different causes, and there is also reason to suppose that some degree of cirrhosis frequently occurs without any symptoms, and never progresses beyond a certain stage. Dr. Saundby drew up a list of ten varieties of hepatic cirrhosis. Besides the alcoholic and biliary, he describes the cardiac, tuberculous, diabetic, malarial, scarlatinal, rachitic, and two forms of syphilitic cirrhosis. To these may be added an enteric type. In some of these, such as the syphilitic and rachitic, the prognosis is more favourable than in others, but in comparing them we notice that under the two first classes we have a somewhat ill-defined group of diseases of varying severity and causation. Certainly, besides cirrhosis produced by obstruction of the common duct, there are other important non-alcoholic forms. Hypertrophic and atrophic are misleading names for classification, since in certain stages of the same disease the liver may be enlarged or diminished. Still, Michell Clarke, in describing minutely a case of the *cirrhose hypertrophique avec ictère* where there was no hypertrophy at the time of observation, notices the rarity of similar recorded cases. The majority of instances of biliary cirrhosis appear as hypertrophic, whether that be because they die before passing into the atrophic stage or no; but atrophic cases of intracellular nature do occur, and may pass into acute yellow atrophy. Hence the prognosis in such cases is more serious than in the ordinary alcoholic form. Dr. Clarke notices the absence of ascites and of enlarged abdominal veins, while jaundice appears early, and is well marked. However, it is probable that these cases may have lasted in a hyperthropic form for a long period, but tend to a rapid termination if they reach the atrophic stage.

Saundby divides cases of hob-nailed liver into those presenting hæmatemesis and those where ascites is prominent, the latter being twice as frequent as the former. Now ascites is prevented by the formation of collateral venous channels, by which the portal blood is conveyed to the inferior vena cava along the coats of the stomach and œsophagus. Here may occur œsophageal varices, which sometimes give way, and even produce fatal results. The importance of this danger has been emphasised of late by Wilson, Kundrat, and others. Where there is cirrhosis present, and slight or vanishing ascites, and especially if sudden, profuse hæmorrhages occur without an admixture with the contents of the stomach, we have strong reason for diagnosing the existence of such varices. The termination may be rapid, but a fatal recurrence has been known as long as twelve years after the first hæmorrhage. As Dr. Wilson has pointed out, there is danger in the employment of ergotin in such cases, while complete rest for the œsophagus, and the administration of nitrate of amyl and hammamelis may be of value. If the collateral circulation is created without failure of the veins, the patient may then survive for long periods. It is stated that one-third of the cases of alcoholic cirrhosis found after death at Guy's Hospital presented no symptoms, and their age actually exceeded that of those who died from the ordinary type of the disease. Under these circumstances, if the patient has a mind to reform his diet, and save the uninjured remnant of his liver, he may prolong his life very considerably. Unfortunately, he has usually very little mind, or, at least, will, left to help himself.

Another danger present in all advanced cases is cholæmia. With the destruction of the liver cells, less and less of its excretory and metabolic functions are performed. Peptones pass through the collateral vessels, and, especially where jaundice appears, products which should have been removed accumulate. A sudden chill or some slight ailment is followed by drowsiness, coma, and death. The irritation of a sudden bout of drinking, or a great mental depression, may at any time bring on a fatal termination. The kidneys are frequently affected, and unable to eliminate the poison. Active purging occasionally succeeds for a time, but we have to consider what are the special poisons concerned, and what remedies exist which cause a rapid excretion or destruction of those substances. Unfortunately, neither the ordinary salines, cholagogues, or intestinal antiseptics can be relied on, nor have we certain knowledge what the poisons always are.

The other great type of alcoholic cirrhosis is marked by ascites. Here early and repeated tapping, with strict attention to diet may considerably prolong life and give relief from most of the symptoms. Dr. Saundby instanced a man who had been thus cured of his ascites six years previously, and remained in fairly good health. Copaiba-resin, and perhaps sugar of milk may for a while replace the tapping, so as to keep down the ascites and give time for the formation of a collateral circulation. By vigorous treatment, and, above all, by abstention from alcohol, many of these patients may be preserved. A milk and fish diet may often be adopted with great benefit, but all these measures have less probability of success when the hypertrophic has passed into an atrophic stage, just as in intracellular cirrhosis. It does not seem that any clear distinction can be made between these two forms as distinct diseases, or as produced by different poisons. The stage of enlargement may be shorter or longer, and may follow the abuse of beer or spirits, but it tends to pass into atrophy and to become more and more intractable to treatment.

In kidney disease we have learnt to recognise that the work of the body may be carried on indefinitely with the residuum of healthy kidney by careful diet, warmth, and the avoidance of chills. In cirrhosis of the liver the evidence is in favour of a similar conclusion, only that it is frequently impossible to get the patient to refrain from drink, and from thus setting up new attacks.

In the non-alcoholic forms of hypertrophy and such cases as those recorded by Dr. Clarke, we have more difficulty in forming a prognosis, except that we see a new danger in the shape of acute atrophy may here crop up. The syphilitic types often yield to remedies such as really large doses of iodide. Nothing is clearer than the almost magical effects of twenty and thirty grain doses of iodide in tertiary lesions when small ones have no effect. Rachitic cirrhosis seems to be also amenable to treatment, but we are not aware that much can be done in tuberculous and malarial cases.

In each type of the disease our prognosis must depend on how far we are able to remove the irritating or obstructing agent (to adopt Mr. Munro Smith's classification); secondly, whether we have enough healthy tissue left to carry on the work if the patient leads a carefully regulated life; and lastly, on whether we can overcome accidental lesions such as hæmorrhage, ascites, or in some forms acute yellow atrophy. We have not yet learned whether the excision of part of a cirrhosed liver would be followed by the growth of healthy tissue on the lines of Pick's experiments, but it seems that, even with our present means, the disease is not so entirely hopeless as it has been thought.

